# Computational Methodology of Optical Molecular Imaging

**DOI:** 10.1155/2013/432107

**Published:** 2013-10-09

**Authors:** Chenghu Qin, Yujie Lu, Jimin Liang, Deming Kong, Huadan Xue

**Affiliations:** ^1^Chinese Academy of Sciences, Beijing 100864, China; ^2^Center for Molecular Imaging, University of Texas Health Science Center at Houston, Houston, TX 77030, USA; ^3^School of Life Science and Technology, Xidian University, Xi'an, Shaanxi 710071, China; ^4^College of Chemistry, Nankai University, Tianjin 300071, China; ^5^Department of Radiology, Union Hospital, Chinese Academy of Medical Sciences and Peking, Union Medical College, Beijing 100730, China

Molecular imaging emerging in the early 21st century has been rapidly developed. Over more than 10 years, optical molecular imaging has become an important tool in preclinical research and has been translated into the clinical for clinical diagnosis and therapy. Computational methodology has shown its indispensable position in developing molecular probes, building imaging instruments, researching reconstruction algorithms, and studying signal and image processing. Relevant research not only adapts existing computational methods but also develops novel strategies to tackle with challenging problems in optical molecular imaging.

This special issue is devoted to the topic of computational algorithms and strategies in optical molecular imaging. Five exciting papers assembled in this special issue present a common theme, that is, improving the imaging and image processing performance in optical molecular imaging by using computational techniques. The papers address several problems in optical molecular imaging, including optical tomographic reconstruction for bioluminescence tomography and image processing such as image segmentation, 3D rendering, and cell morphological measurements. In detail, J. Yu et al. make use of a hybrid multilevel adaptive finite element reconstruction scheme and sparse regularization to acquire quantitative information of bioluminescent sources. The work of Q. Wu et al. is to take advantage of a high-order photon propagation model and Bregman optimization algorithm to improve bioluminescence tomography. The morphology information of living cells and 3D tissue images at the micrometer level can be obtained by using existing computational algorithms from the developed digital holographic microscopy and multichannel spectral imaging laser scanning confocal microscope in the research progress of Y. Wang et al. and Y. Zhang et al., respectively. J. Zheng et al. report that retinal vessels are also efficiently extracted with multiscale hessian-enhancement-based nonlocal mean filter.

